# A rare presentation of CASPR2-associated Morvan syndrome overlapping with GM1-positive AMSAN: a case report

**DOI:** 10.3389/fimmu.2026.1755001

**Published:** 2026-02-11

**Authors:** Yajing Wu, Xiaoli Tang, Tianyuan Guan, Jing Xu, Peiyuan Lv, Yanhong Dong

**Affiliations:** 1 Department of Neurology, Hebei Medical University, Shijiazhuang, Hebei, China; 2 Department of Neurology, Hebei General Hospital, Shijiazhuang, Hebei, China; 3 Department of Neurology, Hebei Provincial Key Laboratory of Cerebral Networks and Cognitive Disorders, Shijiazhuang, Hebei, China

**Keywords:** acute motor and sensory axonal neuropathy, contactin-associated protein-like 2, gangliosides, Guillain-Barré syndrome, Morvan syndrome

## Abstract

**Background:**

Morvan syndrome is a rare autoimmune disorder characterized by peripheral nerve hyperexcitability with autonomic and central nervous system involvement, most commonly associated with antibodies against contactin-associated protein-like 2 (CASPR2). Acute motor and sensory axonal neuropathy (AMSAN) is an axonal variant of Guillain–Barré syndrome linked to anti-ganglioside antibodies and often manifests as severe limb weakness. Their concurrent presentation is unusual and raises the possibility of shared immune targets within peripheral nerve microdomains.

**Case presentation:**

A 70-year-old man presented with a relapsing course of progressive lower-limb weakness accompanied by widespread muscle twitching, severe insomnia with nocturnal hyperarousal, and refractory constipation. He had a prior episode diagnosed as AMSAN that improved after immunotherapy but relapsed four months after treatment was discontinued. Neurological examination demonstrated bilateral lower-limb weakness with reduced tendon reflexes. Moreover, electrophysiological studies confirmed diffuse multifocal peripheral nerve injury with superimposed peripheral nerve hyperexcitability. In addition, immunologic testing revealed serum anti-GM1 antibodies and anti-CASPR2 IgG in both serum and cerebrospinal fluid. Collectively, these findings supported a diagnosis of recurrent AMSAN coexisting with CASPR2-associated Morvan syndrome. Combined immunotherapy with corticosteroids and intravenous immunoglobulin, alongside symptomatic management, resulted in marked clinical improvement.

**Conclusion:**

This case report describes a rare overlap of relapsing AMSAN and Morvan syndrome. This antibody-defined coexistence is hypothesis-generating and may reflect synergistic immune injury involving nodal and paranodal regions. This case underscores the importance of recognizing overlapping phenotypes to guide diagnostic profiling and immunomodulatory therapy.

## 1 Introduction

Acute Motor and Sensory Axonal Neuropathy (AMSAN) is a subtype of Guillain–Barré syndrome (GBS) characterized by the presence of anti-GM1 (monosialotetrahexosylganglioside) and anti-GD1a (disialoganglioside GD1a) antibodies, or other antiganglioside antibodies. GM1 and GD1a are sialylated glycosphingolipids enriched on neuronal membranes—particularly at the node of Ranvier—and they serve as key antigenic targets in axonal GBS variants. Through molecular mimicry, these antibodies target gangliosides on motor and sensory neurons, activate the complement system, and disrupt nodal ion-channel architecture (1). This process leads to conduction failure and axonal degeneration. AMSAN typically presents with acute-onset, symmetrical limb weakness accompanied by prominent sensory disturbances. The disease often progresses rapidly and is associated with a generally poor prognosis (2). In essence, AMSAN reflects axonal injury that compromises excitability-dependent impulse conduction, whereas other autoimmune disorders can also interfere with neural signaling by primarily increasing neuronal excitability, as exemplified by peripheral nerve hyperexcitability syndromes (PNHS). Morvan syndrome (MoS) is a rare autoimmune disorder within the spectrum of PNHS, and is classified among voltage-gated potassium channel (VGKC)-complex antibody–associated disorders. It is most commonly linked to anti-contactin-associated protein-like 2 (CASPR2) antibodies, while anti-leucine-rich glioma-inactivated 1 (LGI1) antibodies may also be present in a subset of cases. The core clinical features include intractable insomnia, autonomic dysfunction (i.e., dysautonomia), and continuous neuromyotonia (3). Although MoS primarily affects the peripheral nervous system (PNS), increasing evidence indicates that VGKC-complex antigens are also expressed in central nervous system (CNS) regions, including the hippocampus, cerebral cortex, cerebellum, brainstem, and hypothalamus. This distribution provides a mechanistic basis for CNS injury. VGKC-complex autoantibodies frequently target CASPR2, a neuron-surface adhesion protein expressed in limbic and other CNS circuits. These antibodies bind extracellular epitopes. They disrupt the CASPR2-associated organization of Kv1 channels. This process alters neuronal network excitability. Consistent with this, early observations in MoS suggested that central symptoms reflect direct antibody effects on the limbic system. Notably, these symptoms were often reversible with plasma exchange. As a result, some patients develop prominent clinical manifestations, including persistent consciousness disturbances, refractory seizures, and a high relapse rate (4). Taken together, although AMSAN and MoS are mediated by distinct antibodies, both represent autoimmune disorders involving the peripheral nervous system. Their coexistence is exceedingly rare. This overlap may indicate shared immune triggers or a more complex mixed phenotype, thereby complicating early differential diagnosis. Furthermore, it remains to be determined whether outcomes in these patients are worse than those in patients with a single entity.

We herein report a case of MoS coexisting with AMSAN. We propose that a possible antibody-mediated pathogenic interplay may exist within peripheral nerves. This case provides a novel perspective for early diagnosis and targeted immunotherapy in overlapping autoimmune neuropathies.

## 2 Case presentation

A 70-year-old male was admitted on April 17, 2025, with progressive lower-limb weakness and widespread muscle twitching for one month’s duration. The patient experienced a flu-like prodrome, and subsequently developed worsening weakness in both lower limbs, necessitating assistance to stand. He also experienced muscle twitching predominantly involving the lips, abdomen, and calves, which presented as visible subcutaneous vermicular movements. In addition, prior to the current admission, he developed prominent insomnia, characterized by subjective hyperarousal at bedtime and sleep onset latency exceeding 1 hour without the use of hypnotic medication. After falling asleep, he experienced frequent early awakenings and fragmented nocturnal sleep. This poor quality of sleep resulted in excessive daytime sleepiness and impaired attention, progressing to an emerging reversal of the sleep–wake cycle. Concurrently, he reported constipation (with no bowel movement for 5 consecutive days prior to admission and no other accompanying gastrointestinal symptoms). No relevant medications had been taken prior to admission.

The patient’s past medical history included poliomyelitis for over 60 years, with residual weakness in the right lower limb, as well as previous lower limb trauma that resulted in bilateral weakness; he ambulated with a cane and lived independently prior to admission.

Approximately six months before the current admission, the patient developed a flu-like prodrome followed by progressive lower-limb weakness. On admission, neurological examination showed proximal strength of 2/5 in both lower limbs, distal strength of 3/5 in the left lower limb, and complete distal paralysis (0/5) in the right lower limb, with reduced tendon reflexes. Cerebrospinal fluid analysis revealed elevated protein with mild pleocytosis (**Table 1**), and serum ganglioside antibody testing was positive for GM1 IgG. These findings supported a diagnosis of AMSAN. He was treated with intravenous immunoglobulin (IVIG) at 0.4 g/kg/day for 5 consecutive days, improved clinically, and was discharged. Two weeks after discharge, he discontinued all medications and did not attend the scheduled follow-up visits.

**Table 1:** CSF findings at initial presentation and relapse.

Category	Parameters	2024-10-31	2025-04-21
Cerebrospinal fluid (CSF) routine examination	Appearance	clear and colorless	clear and colorless
	Pandy reaction	Weakly positive	negative
	Nucleated cell count (×10^6/L)	22	7
	White blood cell count (×10^6/L)	22	7
	Red blood cell count (×10^9/L)	0	0
	Mononuclear cell count (×10^6/L)	21	6
	Mononuclear cells (%)	95.5	85.7
	Polymorphonuclear cell count (×10^6/L)	1	1
	Polymorphonuclear cells (%)	4.5	14.3
	Eosinophil count (×10^6/L)	0	0
	High-fluorescence cell count (×10^6/L)	0	0
	High-fluorescence cells (/100 WBC)	0	0
CSF biochemistry	Chloride (mmol/L)	121.2	126.9
	Glucose (mmol/L)	3.19	3.53
	Total protein (mg/dL)	95.55	16.13
CSF cytology	Lymphocytes(%)	74	93
	Eosinophils(%)	0	1
	Monocytes(%)	23	5
	Neutrophils(%)	1	1
	Basophils(%)	1	0
	Activated monocytes(%)	1	0
	Red blood cells(%)	0	scattered distribution

The initial CSF analysis was characterized by predominant protein elevation accompanied by mild pleocytosis, suggesting an immune-mediated abnormality. In contrast, the second analysis revealed a mild, lymphocyte-predominant pleocytosis, consistent with a low-grade inflammatory response within the central nervous system.

Upon the current admission, his vital signs were as follows: temperature 36.2°C, pulse 85 bpm, blood pressure 144/90 mmHg, and respiratory rate 20 breaths/min. Neurological examination showed normal muscle strength (Grade 5) in both upper limbs. In the lower limbs, muscle strength was Grade 2 proximally and Grade 3 distally on the left, and Grade 2 proximally with complete distal paralysis (Grade 0) on the right. Tendon reflexes were bilaterally diminished.

**Figure 1:**
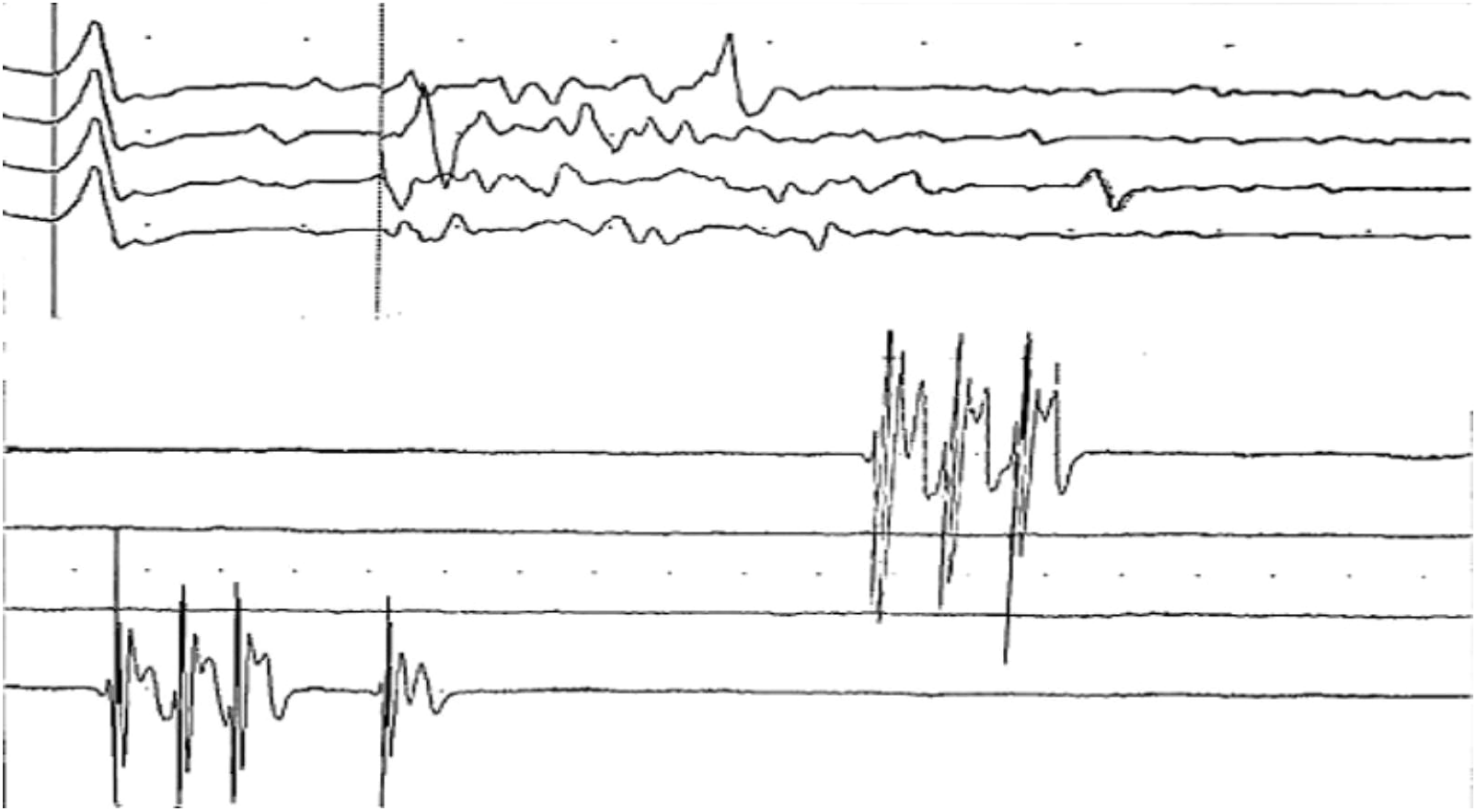
Electrophysiological findings demonstrating peripheral nerve hyperexcitability. After discharges are observed in the upper trace, while spontaneous discharges are seen in the lower trace.

**Figure 2:**
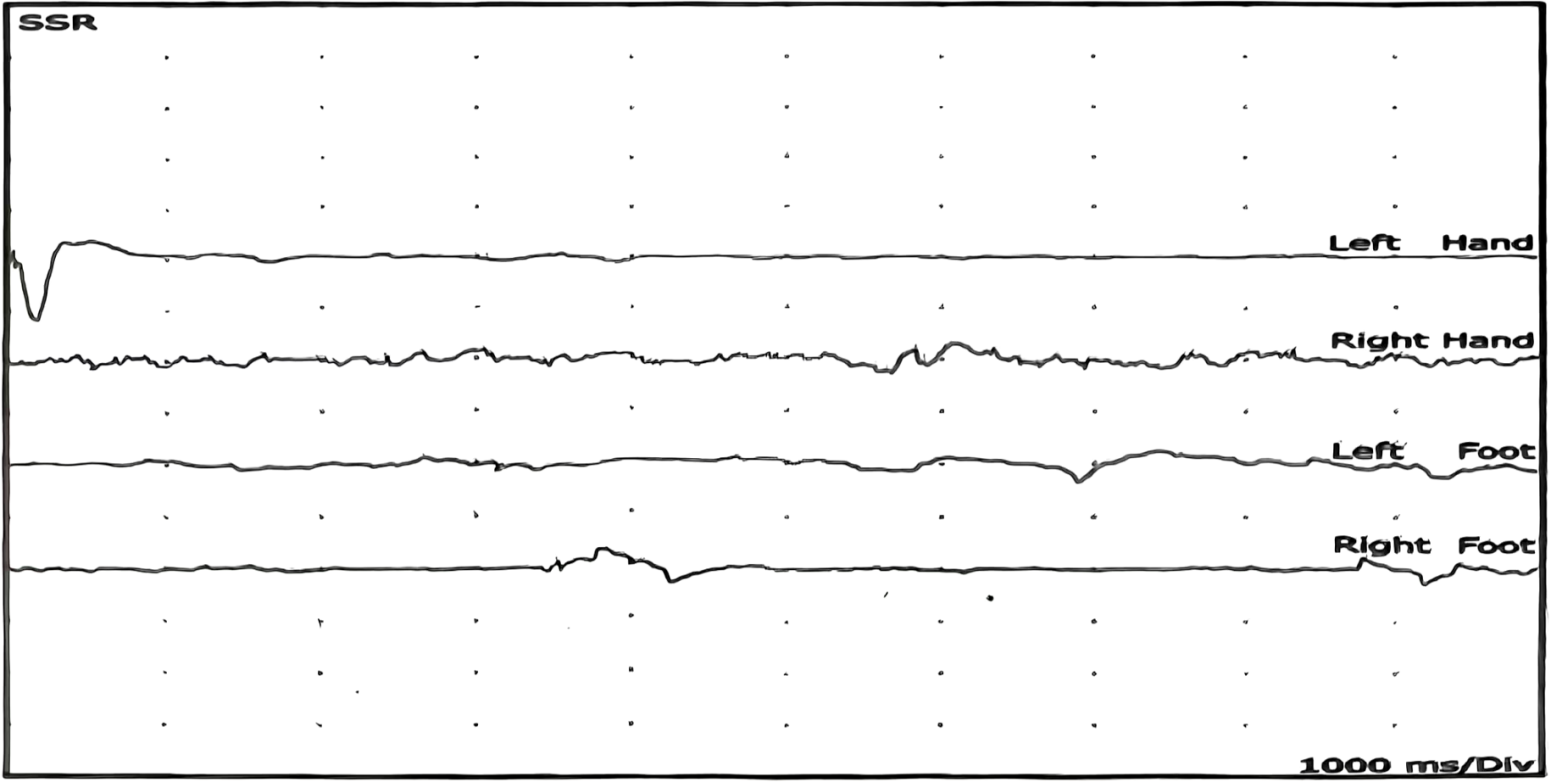
Sympathetic skin responses (SSR) testing. Waveforms recorded from the palms and soles showing no response (NR) in all four limbs, indicating severe autonomic dysfunction. NR, no response; SSR, sympathetic skin response.

On admission, his Pittsburgh Sleep Quality Index (PSQI) score was 19. The PSQI is a validated self-report questionnaire that assesses sleep quality and disturbances over the past month and yields a global score ranging from 0 to 21, with higher scores indicating poorer sleep quality. Investigations showed no evidence of thymoma on chest CT. Serum potassium was 3.3 mmol/L. Serum immunoglobulin G was elevated at 20.17 g/L (reference range: 7.0-16.0 g/L), and interleukin-2 receptor alpha (IL-2RA) was markedly elevated at 1340.89 pg/mL, indicating T-cell activation and a concomitant B-cell response. A stool routine examination was unremarkable except for a weakly positive fecal occult blood test. No stool culture, serological testing for *Campylobacter jejuni*, or other pathogen-specific investigations were performed at that time. Multifocal peripheral neuropathy was demonstrated on electrophysiological examination (**Table 2**), with F-wave after discharges and spontaneous fibrillation potentials at rest (**Figure 1**), and abnormal sympathetic skin responses in all four limbs (**Figure 2**).

**Table 2:** Nerve conduction study findings demonstrating asymmetric axonal involvement.

Tested nerve	Amplitude(μV)	Contralateral amplitude (μV)	Conduction velocity(m/s)	Contralateral velocity (m/s)	Latency(ms)
Right median nerve (motor)					
Wrist	5.0	10.7	-	-	4.8
Elbow	3.9	10.7	60.3	56.9	8.2
Right median nerve (sensory)	5.6	12.3	43.8	53.7	3.2
Right common peroneal nerve (motor)					
Ankle	3.7	7.7	-	-	3.0
Fibular head	3.6	6.5	49.1	49.2	8.7
Left superficial peroneal nerve (sensory)	1.7	30.8	39.6	57.0	2.4
Right tibial nerve (motor)					
Ankle	4.2	6.3			3.1
Knee	2.9	5.2	47.1	44.6	10.0

Prolonged distal latency and reduced amplitude were observed in the right median motor nerve compared to the contralateral side. The right median sensory nerve showed decreased amplitude and slowed conduction velocity. The right ulnar sensory nerve exhibited reduced amplitude. The amplitude of the right common peroneal motor nerve was lower than that of the contralateral side. The left superficial peroneal sensory nerve demonstrated reduced amplitude and decreased conduction velocity. The right tibial motor nerve showed decreased proximal amplitude.

**Figure 3:**
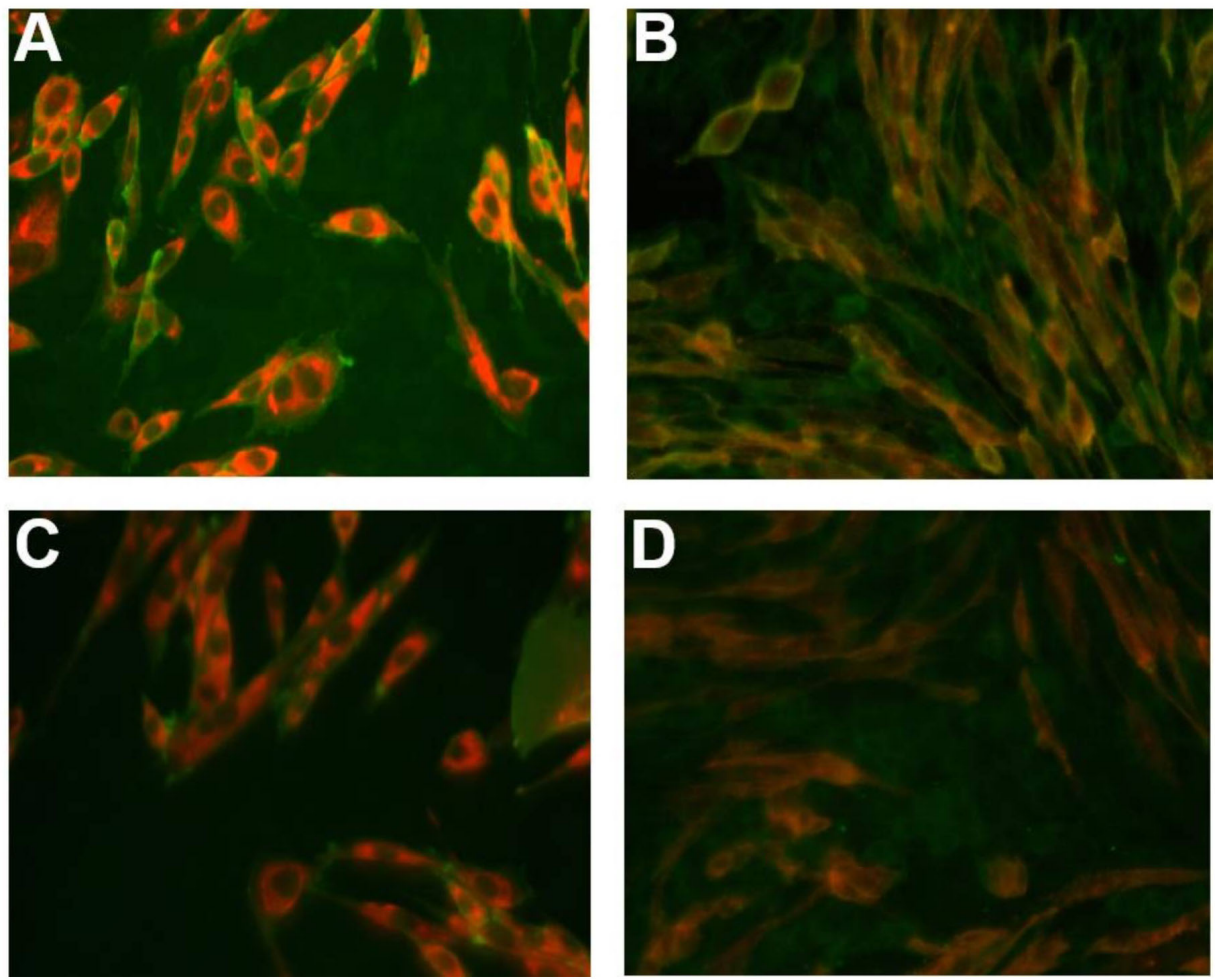
Positive detection of anti-CASPR2 IgG antibodies in serum and cerebrospinal fluid by cell-based assay. **(A, B)** Serum tested positive for CASPR2-IgG antibodies with a titer of 1:320; **(A)** shows the positive control. **(C, D)** CASPR2-IgG antibodies were detected in cerebrospinal fluid at a titer of 1:1; **(C)** represents the positive control.

A lymphocytic response was demonstrated in CSF cytology with scattered fresh red blood cells (**Table 1**). Immunologic testing (**Figure 3**) revealed serum positivity for anti-GM1 IgM antibodies (immunoblot, qualitative), anti-CASPR2 IgG (cell-based assay, titer 1:320), and elevated voltage-gated potassium channel (VGKC)-complex antibodies (radioimmunoassay, 160.29 pmol/L). Conversely, anti-LGI1 IgG and other relevant autoimmune antibodies were negative. The CSF was also positive for anti-CASPR2 IgG (cell-based assay, titer 1:1). CSF biochemistry, routine analysis, and anti-double-stranded DNA testing were unremarkable.

The patient was admitted with recurrent worsening lower-limb weakness accompanied by constipation, insomnia, and widespread muscle twitching. Importantly, there was a clear period of clinical remission before this relapse, which favored a recurrent acute neuropathy rather than chronic inflammatory demyelinating polyradiculoneuropathy (CIDP). On examination, muscle bulk was unchanged compared with previous assessments, tendon reflexes were reduced, and the disease course of this episode was less than four weeks. Together with serum anti-GM1 positivity and nerve conduction studies showing combined motor and sensory peripheral nerve involvement, these findings were consistent with AMSAN. In parallel, the patient had prominent muscle fasciculations, severe insomnia, and constipation. Electrophysiology further demonstrated after discharges and spontaneous activity, and antibody testing revealed VGKC-complex antibodies with anti-CASPR2 positivity. Taken together, these clinical, electrophysiological, and immunologic features supported the diagnosis of MoS coexisting with recurrent AMSAN. Immunomodulatory therapy included corticosteroids and IVIG, along with Gabapentin and Carbamazepine to manage neuronal hyperexcitability. Symptomatic treatments such as laxatives and sedative agents were also administered. The patient exhibited marked clinical improvement and was discharged after an eight-day of hospitalization; at discharge, the previously prominent muscle twitching around the lips had resolved, and only rare, barely visible muscle twitching remained in the abdominal wall and calves. Muscle strength was MRC grade 5/5 in both upper limbs, left proximal strength had improved to grade 3/5, and grade 2/5 proximally with 0/5 distally in the right lower limb.

Following discharge, the patient continued immunosuppressive therapy with intravenous methylprednisolone at 250 mg once daily for 5 days, then tapered to 120 mg daily for another 5 days, and subsequently transitioned to oral prednisone 40 mg. Gabapentin was prescribed at 600 mg three times daily. Mycophenolate mofetil was initiated at 0.25 g twice daily and escalated to 0.5 g twice daily after one week. At the 6-month follow-up after discharge, no relapse had occurred. According to his family, he could hold objects steadily with both hands, walk with the aid of a walker, had no observable muscle twitching, and showed a Pittsburgh Sleep Quality Index (PSQI) score of 4, demonstrating a favorable long-term recovery.

## 3 Discussion

This study reports a rare case of overlapping autoimmune neurological disorders, initially manifesting as progressive bilateral lower limb weakness and widespread muscle fasciculations, ultimately diagnosed as recurrent AMSAN coexisting with MoS. A review of the literature reveals no previously reported cases of comorbidity between these two entities. Recognition of such cases is essential for advancing our understanding of the immunopathological involvement of the nodes of Ranvier and their associated molecular structures in autoimmune neuropathies.

AMSAN is an axonal variant of Guillain–Barré syndrome primarily mediated by autoantibodies against GM1 or GD1a gangliosides (5). These autoantibodies bind to gangliosides in the axolemma at the nodes of Ranvier, disrupting nodal integrity and leading to sensory abnormalities, hyporeflexia or areflexia, and symmetrical limb weakness (6). In this case, anti-GM1 antibodies were detected in the patient’s serum, which target the nodes and paranodes of Ranvier, thereby impairing nerve conduction. This resulted in progressive bilateral lower limb weakness, with electrophysiological findings indicating involvement of both sensory and motor fibers. A limitation of this report is the lack of specific titer or quantitative value for the serum GM1-IgM antibody. Since antibody titers often correlate with disease activity and severity in autoimmune neuropathies, this limits our ability to fully assess the immune response’s role in the disease pathogenesis. However, the clear qualitative positivity, combined with the patient’s typical AMSAN clinical presentation and electrophysiological findings, is sufficient to support the diagnosis of an anti-GM1-associated neuropathy.

In parallel, MoS represents a VGKC-complex antibody–associated disorder in which immune-mediated dysfunction may extend beyond nodes to involve paranodal and broader neuro-axis targets. MoS is a rare mixed central and peripheral autoimmune encephaloneuropathy typically associated with antibodies against components of the VGKC complex, primarily CASPR2 and LGI1 (7). In our case, CASPR2 antibodies were detected. CASPR2 is normally expressed in the hippocampus, cerebral cortex, cerebellum, and brainstem, as well as in autonomic fibers and the paranodal regions of peripheral nerves (8). The widespread binding of autoantibodies to these normally distributed targets underlies the diverse clinical phenotype of MoS. The patient exhibited clinical signs consistent with dysregulation of both action potential initiation and repolarization processes, indicating a complex imbalance in neuronal excitability modulation.

**Figure 4:**
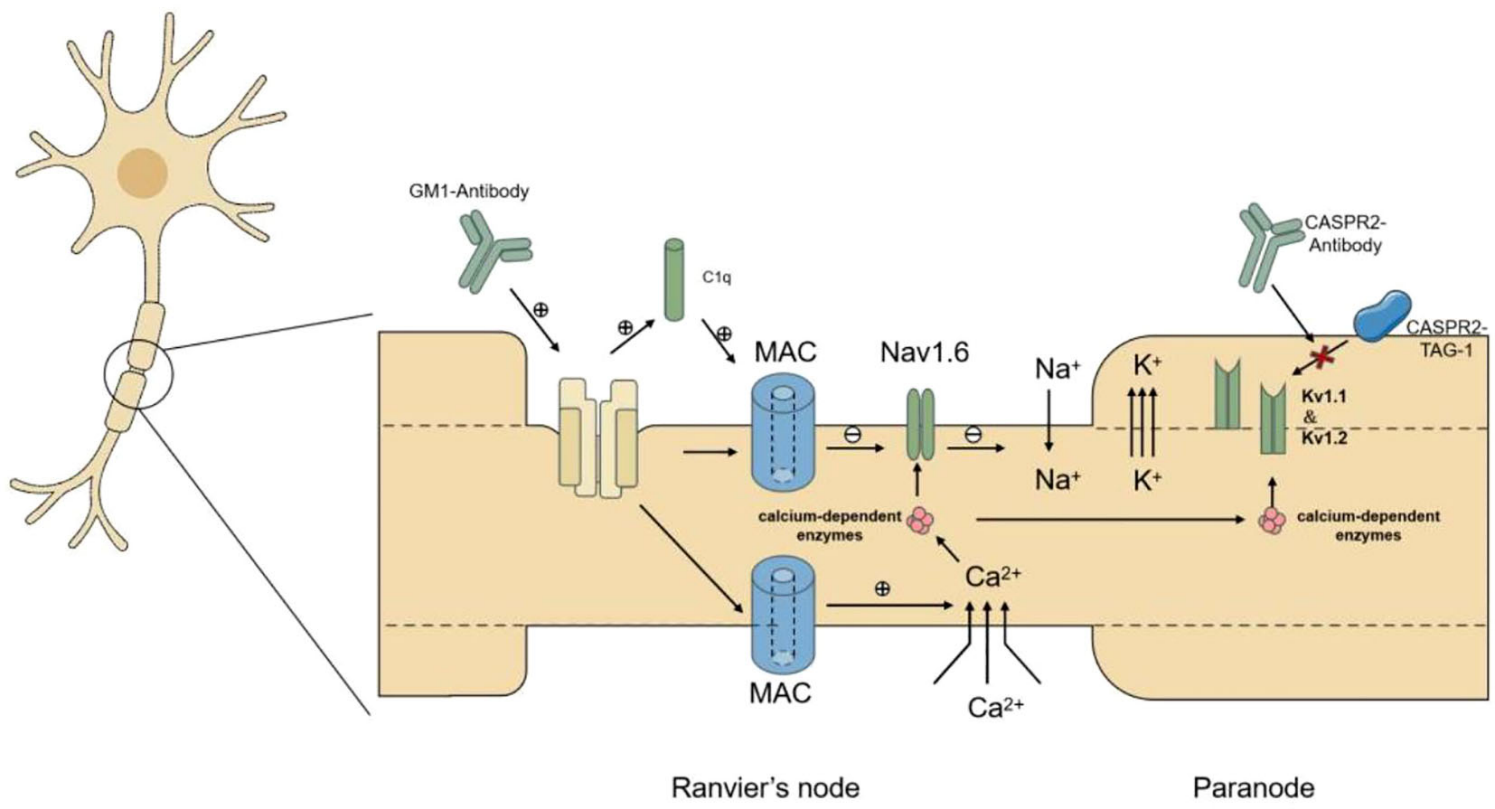
Immune-mediated nodal and paranodal dysfunction. Schematic representation of nodal and paranodal immune injury mediated by anti-GM1 and anti-CASPR2 antibodies, illustrating the disruption of Nav1.6 and Kv1 channel localization and the resulting bidirectional imbalance in action potential initiation and repolarization. CASPR2, contactin-associated protein-like 2; GM1, ganglioside M1; Kv1, voltage-gated potassium channel subfamily 1; MAC, membrane attack complex; Nav1.6, voltage-gated sodium channel 1.6.

Taken together, the co-detection of anti-GM1 and anti-CASPR2 antibodies suggests a nodal–paranodal continuum of immune involvement, providing the rationale for the following conceptual framework. Based on the immunopathological findings, we propose the theoretical model of “Nodal-paranodal cross-immune injury,” synergistically mediated by anti-GM1 and anti-CASPR2 antibodies (**Figure 4**). Anti-GM1 antibodies specifically target gangliosides embedded in the axolemma at the nodes of Ranvier. After complement activation, these antibodies can trigger formation of the membrane attack complex (MAC, C5b-9), which damages the nodal axolemma and disrupts Nav1.6 channel clustering and function, ultimately leading to conduction failure and impaired nerve impulse transmission (9). Anti-CASPR2 antibodies, predominantly of the IgG4 subclass, target the paranodal region where they disrupt the CASPR2-TAG-1 adhesion complex, thereby impairing the membrane anchoring of Kv1.1/Kv1.2 potassium channels essential for axonal excitability (10). This leads to mislocalization, reduced expression, or internalization of these channels, weakening their role in dampening neuronal excitability and contributing to local hyperexcitability. The concurrent attack on both the nodal and paranodal compartments disrupts generation and repolarization of action potentials necessary for saltatory conduction, forming a dual-axis pathophysiological imbalance. Studies indicate that MAC insertion alters membrane permeability, triggering calcium influx and activating calcium-dependent enzymes (11). These enzymes may degrade anchoring proteins, disrupt sodium channel localization, and destabilize lipid microdomains essential for Kv1 channels. However, this proposed model remains hypothetical, as experimental evidence directly supporting synergistic injury by these antibodies is lacking. Collectively, these mechanisms could theoretically amplify the pathogenic synergy between anti-GM1 and anti-CASPR2 antibodies.

These three dynamically imbalanced immune mechanisms push nerve impulse transmission toward a pathological state characterized by both conduction vulnerability and hyperexcitability. This state is reflected in the patient’s clinical profile of failed motor conduction with concurrent fasciculations. The recently proposed concept of “nodo-paranodopathy spectrum disorders” reflects this integrated pathophysiological mechanism, highlighting the nodal-paranodal region as a pathological focal point for the comorbidity of various antibody-mediated neuropathies (12). However, this “nodal–paranodal cross-immune injury” model remains a hypothesis derived from correlative clinical and immunologic data in a single patient; in the absence of *in vitro* or *in vivo* experimental evidence directly demonstrating synergistic damage by these antibodies, it should be regarded as a theoretical framework rather than a proven mechanism. Within this framework, electrophysiology translated molecular-level injury into quantifiable abnormalities. The M-wave or compound muscle action potential (CMAP) reflects motor axonal integrity, while the F-wave assesses proximal conduction along the full axonal length. In our patient, disruption of Nav1.6 sodium channels manifested as reduced CMAP amplitudes and absent F-waves. These findings are consistent with impaired impulse propagation and reduced recruitment of motor neurons in the F-wave pathway. Conversely, disrupted localization of Kv1 potassium channels was evidenced by after discharges following M-waves. These after discharges are repetitive responses that follow the initial CMAP. They are widely regarded as markers of peripheral nerve hyperexcitability, reflecting delayed repolarization (13). These electrophysiological abnormalities effectively render the pathophysiology of this acquired channelopathy evident and furnish reliable metrics for clinical evaluation of action potential threshold, action potential amplitude, and action potential repolarization.

In this case, a preceding infection was noted at disease onset, raising the possibility of a classical triggering factor, such as Campylobacter jejuni, for GBS and its axonal variant AMSAN. The bacterial lipooligosaccharides share molecular mimicry with ganglioside GM1, leading to the generation of anti-GM1 antibodies and complement activation via molecular mimicry, ultimately causing AMSAN (14). Although there is currently no definitive evidence linking C. jejuni to MoS, infections are recognized antecedent triggers for post-infectious autoimmune neuropathies. Several pathogens including Campylobacter jejuni, Mycoplasma pneumoniae, cytomegalovirus, Epstein–Barr virus, influenza, hepatitis E, Zika and dengue are reported for GBS (15). Whether C. jejuni acts as a dual sensitizer or whether there is a shared antigen-presenting cell-mediated immunological pathway remains to be elucidated. A further limitation is that, despite the antecedent diarrheal illness suggestive of a possible Campylobacter jejuni trigger, no stool culture or serological testing for C. jejuni or other pathogens was performed, so a direct infectious link cannot be confirmed in our study.

Given the overlapping presentation and potential diagnostic uncertainty, a structured diagnostic approach is particularly important. Although AMSAN and MoS represent distinct and mutually exclusive phenotypes regarding target antigens and pathogenesis, our analysis indicates overlapping clinical manifestations and immunological characteristics. This overlap manifests as a compound phenotype featuring both axonal damage and nerve hyperexcitability symptoms. Clinically, the critical step in diagnosing this compound phenotype involves electrophysiological assessment. This assessment focuses first on axonal conduction velocities and spontaneous electromyographic activity, and it is complemented by nerve ultrasonography, which can detect axonal degeneration and structural changes at the nodes and paranodes of Ranvier. Antibody profiling should also be performed, including ganglioside antibodies and VGKC-spectrum antibodies. With these elements considered together, clinicians can avoid misdiagnosis or delayed diagnosis as a single phenotype.

Once an overlap phenotype is recognized, management should address both immune-mediated axonal injury and antibody-associated hyperexcitability. Mechanistically, the dual-antibody-positive state drives a multi-target immune response. This process is amplified by inflammatory cytokines (such as IFN-γ and IL-7) and leads to the disruption of the blood–nerve barrier (16). Concurrent triggers—such as thymoma or heavy metal exposure—have been confirmed to initiate both conditions simultaneously, suggesting that therapeutic strategies should prioritize coordinated, multi-target immune interventions (17). Research has demonstrated that a triple-combination immunotherapy approach effectively improves both motor deficits and nerve excitability symptoms (18). IVIG or plasmapheresis can mitigate axonal injury and directly delay disease progression; corticosteroids suppress pro-inflammatory cytokine expression, alleviating the neuroimmune axis’ “inflammation-barrier activation-channel antibody infiltration” pathway, thus improving nerve excitability symptoms and autonomic dysfunction; and B-cell-targeted therapies can directly reduce pathological antibody production.

Therefore, constructing large-scale patient cohorts is urgently needed to clarify the actual comorbidity rate and typical clinical course of these conditions. Establishing dual-antibody animal models could elucidate precise intervention mechanisms, driving the development of dual-target screening and treatment guidelines and optimizing diagnostic and therapeutic processes as well as prognostic management for this complex phenotype.

In conclusion, to the best of our knowledge, we present the first reported case of recurrent AMSAN coexisting with MoS. By exploring the key role of nodal and paranodal structures in autoimmune neuropathies, this case underscores its relevance as a shared immunopathological target and advocates for its clinical recognition to inform early diagnosis and therapeutic strategies.

## Data Availability

The original contributions presented in the study are included in the article/supplementary material. Further inquiries can be directed to the corresponding author/s.
